# Going beyond the current neuroinformatics infrastructure

**DOI:** 10.3389/fninf.2015.00015

**Published:** 2015-06-16

**Authors:** Xi Cheng, Daniel Marcus, John D. Van Horn, Qian Luo, Venkata S. Mattay, Daniel R. Weinberger

**Affiliations:** ^1^Lieber Institute for Brain Development, Johns Hopkins Medical CampusBaltimore, MD, USA; ^2^Department of Radiology, Washington University School of MedicineSt. Louis, MO, USA; ^3^Laboratory of Neuro Imaging, Department of Neurology, Institute of Neuroimaging and Informatics, University of Southern CaliforniaLos Angeles, CA, USA; ^4^Center for Military Psychiatry and Neuroscience Research, Walter Reed Army Research InstituteSilver Spring, MD, USA^†^

**Keywords:** neuroinformatics, infrastructure, neuroscience, informatics, data sharing

The enormous volume of multi-modal neuroimaging data across different neuroscience research communities poses a daunting challenge to traditional methods of data sharing, data archiving, data processing, and data analysis (Van Horn and Toga, 2014).

Neuroinformatics plays a crucial role in creating advanced methodologies and tools for the handling of varied and heterogeneous datasets in order to better understand the structure and function of the brain. These tools and methodologies not only enhance data collection, analysis, integration, interpretation, modeling, and data dissemination, but also promote data sharing and collaboration (Cox, 1996; Smith et al., 2004; Friston, 2006; Marcus et al., 2007; Dinov et al., 2009; Van Horn and Toga, 2009) which are essential elements for making progress efficiently in this rapidly burgeoning field.

The purpose of this special issue is to use case studies of the state-of-art neuroinformatics infrastructure to anticipate and project future generation systems.

A number of leading research groups from different parts of the world were invited to participate in this research topic. Each of the contributions provided a showcase solution to domain specific challenges we currently face. We will try to review these articles according to the categories of the issues they covered. Some articles covered multiple categories. However, due to the limited space, we only discuss them under one category.

Articles by Bartsch et al. (2014), Goscinski et al. (2014), Haselgrove et al. (2014), King et al. (2014), Marenco et al. (2014), Muehlboeck et al. (2014), Rane et al. (2014), Rautenberg et al. (2014), Sherif et al. (2014), and Wood et al. (2014), present solutions for data archiving and related issues including, how to efficiently collect, store, query, visualize and share large volume neuroimaging data. Some of these systems are large in scale, geographically distributed, and already have a large dataset and a well-established user community.

Beyond neuroimaging, Sobolev et al. (2014) present a data management platform for neurophysiological data, and Mouček et al. (2014), and Tripathy et al. (2014) describe techniques and methodologies for collecting and managing electrophysiological data.

Once the incoming data have been archived, there are many other important issues that need to be addressed.

First, how to visualize the data to meet domain-specific needs is still an open-ended research question. Gutman et al. (2014) present a light framework to visualize DICOM images stored in the Extensible Neuroimaging Archive Toolkit (XNAT). Hänel et al. (2014) describe an application with two designs for the 3D visualization of the human brain.

Second, how to efficiently process huge volumes of datasets is challenging especially when bottom-up explorative data analysis becomes more and more popular. Contributions from Andronache et al. (2013), Da Mota et al. (2014), Dinov et al. (2014), Eklund et al. (2014), Friedel et al. (2014), and Mahmud et al. (2014), discuss opportunities and methodologies that facilitate large-scale parallel data processing tasks under a heterogeneous computational environment.

Third, how to mine the data i.e., how to extract meaningful information from the data, is the most challenging part of all. Liu and Calhoun (2014) provide a review of multivariate analyses approaches in Imaging Genetics. Goh et al. (2014) discuss challenges in neuroinformatics of Traumatic Brain Injury neuroimaging analysis in the context of structural, connectivity, and functional paradigms. The manuscript by Miller et al. (2013) describes novel neuroinformatics technologies at 1 mm anatomical scale based on high-throughput 3D functional and structural imaging technologies of the human brain. Xiang et al. (2014) explored novel data analysis methodologies and platforms for handling large volumes of neuromagnetic data with a very wide range of temporal frequencies. Kauppi et al. (2014), introduce a versatile software package for inter-subject correlation based analyses of fMRI data.

Finally, there are a number of contributions discussing other topics important to the neuroinformatics infrastructure. Zaslavsky et al. (2014) describe a prototype implementation of digital atlasing infrastructure initiated by the International Neuroinformatics Coordinating Facility (INCF). Herrick et al. (2014) showcase how to use dictionary service to extend metadata across XNAT database instances. Sarwate et al. (2014) review the relevant literature on differential privacy, a framework for measuring and tracking privacy loss in these settings, and demonstrate the feasibility of using this framework to calculate statistics on data distributed at many sites while still providing privacy. Das et al. (2014) report a case study on how to foster discussion and communication by using an open-source content management system. Evans and Polavaram (2013) provide a general commentary article in the field of computational models of biologically realistic neuronal networks.

We intend this Special Issue as more than a compendium of current systems. We wish to stimulate on-going discussions at the level of the neuroinformatics infrastructure including: –what are the common challenges the next generation of infrastructure will have to address? –what new technologies will be of maximum benefit? –how will we go beyond the limits of the current generation infrastructure? and –what are the key features next generation infrastructure should implement? Such discussions will inspire and help guide the development of a state of the art, highly-efficient neuroinformatics infrastructure. Such research community wide productive catalytic reactions will be a testament to the worthiness of our efforts in creating this Special Issue.

## Conflict of interest statement

The authors declare that the research was conducted in the absence of any commercial or financial relationships that could be construed as a potential conflict of interest.
